# Ancestry‐specific studies on Alzheimer’s disease using iPSC‐derived microglia

**DOI:** 10.1002/alz.089416

**Published:** 2025-01-03

**Authors:** Sofia Moura, Luciana Bertholim Nasciben, Jihanne J Shepherd, Lauren Coombs, Aura M Ramirez, Shaina A. Simon, Derek Van Booven, Joe Rivero, Brooke A. DeRosa, Patrice L. Whitehead, Larry D. Adams, Takiyah D. Starks, Pedro R. Mena, Maryenela Z. Illanes‐Manrique, Sergio J. Tejada, Goldie S. Byrd, Mario Cornejo‐Olivas, Briseida E. Feliciano‐Astacio, Farid Rajabli, Liyong Wang, Anthony J. Griswold, Juan I Young, Margaret A. Pericak‐Vance, Derek M. Dykxhoorn, Jeffery M. Vance

**Affiliations:** ^1^ John P. Hussman Institute for Human Genomics, Miller School of Medicine, Miami, FL USA; ^2^ John P. Hussman Institute for Human Genomics, University of Miami Miller School of Medicine, Miami, FL USA; ^3^ John P. Hussman Institute for Human Genomics, University of Miami Miller School of Medicine, Miami, FL, USA, Miami, FL USA; ^4^ John P. Hussman Institute for Human Genomics, Miami, FL USA; ^5^ Maya Angelou Center for Health Equity, Wake Forest University School of Medicine, Winston‐Salem, NC USA; ^6^ Neurogenetics Research Center, Instituto Nacional de Ciencias Neurológicas, Lima Peru; ^7^ Universidad Central del Caribe, Bayamón, PR USA

## Abstract

**Background:**

In the US, African Americans (admixed with African and European) followed by Hispanics (admixed with Amerindian, African, and European) are the most affected groups compared to non‐Hispanic Whites (NHW). While genetic diversity and admixture play crucial roles in disease risk, the ancestry‐specific mechanisms remain poorly understood with most AD‐related studies focusing on NHW. Despite the recent field efforts to include genetically admixed populations, there continues to be a lack of functional studies in AD across the different cell types in these populations. Given the importance of Microglia in AD, we here characterize the genetic regulatory architecture (GRA) on iPSC‐derived Microglia (MGL) in African and Amerindian genomes.

**Method:**

iPSC lines derived from controls and AD patients with >90% genomic content from different ancestries (Amerindian, African, and European) were differentiated into MGL. We performed bulk RNA‐seq and ATAC‐seq, followed by differential expression and accessibility analyses to study the GRA of these admixed populations and its contributions to AD.

**Result:**

We identified 1,103 differentially expressed genes (DEGs) and 267 differentially accessible genes (DAGs) across ancestries. We observed the most differences on both chromatin accessibility and gene expression levels between AI and AF. On the chromatin level and in the context of AD, we observed 2 DAGs (*PRDM7* and *SCIMP*) between AI and AF, and 1 DAG between AI and EU (*PRDM7*). In addition, we identified 10 AD‐risk modifying genes that are differentially expressed between AI and AF ancestries (*ABI3, CTSB, JAZF1, MS4A6A, PILRA, PLEKHA1, RASGEF1C, SORL1, TREM2*, and *TREML2*) and 3 DEGs between AI and EU (*JAZF1, MS4A6A*, and *SORL1*). We identified several DEGs to be involved in lipid metabolism, cholesterol biosynthesis and metabolism, lysosomal activity, and immune response ‐ all highly relevant processes in AD pathology.

**Conclusion:**

We provide new insights into ancestry‐specific genetic risk factors in AD pathophysiology. Here, we report novel transcriptomic and chromatin accessibility data in microglia of AI and AF ancestries that potentially contribute to a differential genetic risk in AD in the different ancestries. Interestingly, those ancestries with greatest migratory differences revealed the largest DEG.

